# TEDC2 correlated with prognosis and immune microenvironment in lung adenocarcinoma

**DOI:** 10.1038/s41598-023-32238-8

**Published:** 2023-03-27

**Authors:** Likui Fang, Wenfeng Yu, Pengfei Zhu, Guocan Yu, Bo Ye

**Affiliations:** Department of Thoracic Surgery, Affiliated Hangzhou Chest Hospital, Zhejiang University School of Medicine, Hangzhou, 310003 China

**Keywords:** Cancer, Genetics, Immunology

## Abstract

Tubulin epsilon and delta complex 2 (TEDC2) is a protein coding gene whose functions are poorly identified yet. This study aimed to identify the role of TEDC2 in prognosis and immune microenvironment of lung adenocarcinoma (LUAD). Through The Cancer Genome Atlas (TCGA) and Gene Expression Omnibus (GEO) databases, the mRNA expression of TEDC2 was upregulated in LUAD tissues compared to normal tissues. The protein level of TEDC2 was also higher in LUAD in the Human Protein Atlas. The receiver operating characteristic (ROC) curve showed that high TEDC2 level could distinguish LUAD patients from normal subjects. In addition, the impact of TEDC2 expression on prognosis was evaluated by Kaplan–Meier and Cox regression analyses, and the results suggested that high TEDC2 expression was significantly associated with poor prognosis and was the independent prognostic factor in LUAD. GO and KEGG pathway analyses indicated the co-expressed genes of TEDC2 were mainly related to mitotic cell cycle processes. Importantly, high expression of TEDC2 indicated low infiltration of immune cells, especially dendritic cells and B cells. TEDC2 was also positively correlated with immune checkpoints such as PDCD1, LAG3 and CD276. Taken together, this study preliminarily revealed the clinical significance of TEDC2 in LUAD and provided novel insights into the role of TEDC2 in immune microenvironment.

## Introduction

Lung cancer is one of the most frequently diagnosed cancer and remains the major cause of cancer-related deaths worldwide^1,2^. More than 80% of total diagnoses were NSCLC, and lung adenocarcinoma (LUAD) is the most common subtype within NSCLC classifications^3^. The 5-year survival rate of NSCLC highly depends on the stage, with roughly 80% in stage I, only 13–60% in stage II to stage III and less than 10% in metastatic disease^4^. Although the standard of care for early-stage NSCLC is still surgical resection, the treatment of advanced or metastatic diseases has undergone remarkable changes during the past decade due to the advances in molecular targeted therapy and immunotherapy^5–7^. However, because of targeted therapy restricted to LUAD containing driver mutations and responses from immunotherapy occurring uncommonly, only a minority of patients benefit from these therapies^8^. Acquired resistances are also the major challenge of both therapies^9^. Therefore, detection of novel biomarkers is crucial for further therapeutic advances.

Tubulin epsilon and delta complex 2 (TEDC2), also named Chromosome 16 open reading frame 59 (C16orf59), is a protein coding gene whose functions are poorly identified yet. Previous studies reported that TEDC2 could highly express in central nervous system lymphoma and might contribute to the tumorigenesis of LUAD^10,11^, but the prognostic and immune features of TEDC2 in LUAD have not been comprehensively characterized. Therefore, this study extracted LUAD samples from The Cancer Genome Atlas (TCGA) and Gene-Expression Omnibus (GEO) databases to identify the diagnostic and prognostic potential of TEDC2, and characterize the association between TEDC2 and immune microenvironment of LUAD via the application of various algorithms. Our bioinformatics analysis would provide a novel insight into the immune features of TEDC2 and reveal the therapeutic potential of targeting TEDC2 in LUAD.

## Material and methods

### Data acquisition

A total of 535 LUAD samples and their clinicopathologic characteristics were downloaded from The Cancer Genome Atlas (TCGA) database (https://portal.gdc.cancer.gov/) and analyzed in this study. The datasets (GSE18842, GSE7670, GSE27262 and GSE140797) were obtained from Gene Expression Omnibus (GEO) database (https://www.ncbi.nlm.nih.gov/gds) to verify the differential expression of TEDC2 between LUAD and normal tissues. The protein levels of TEDC2 between LUAD and normal tissues were compared in the Human Protein Atlas (https://www.proteinatlas.org/) which provides protein immunohistochemistry in normal human tissues and tumor tissues.

### Co-expression gene analysis

The co-expression genes positively and negatively correlated with TEDC2 expression were explored through the LinkFinder module in the LinkedOmics database (http://www.linkedomics.org/login.php), which is a web portal that analyzes multi-omics data from TCGA datasets^12^. LinkInterpreter module was used to perform the gene set enrichment analyses (GSEA) of Gene Ontology biological process (GO_BP) and Kyoto Encyclopedia of Genes and Genomes (KEGG) pathways.

### Correlation between TEDC2 and immune microenvironment

The assessment of immune cell infiltration was conducted by the single-sample gene set enrichment analysis (ssGSEA) which contained 28 immune cells^13,14^. The infiltration of stromal and immune cells was further assessed by Estimation of STromal and Immune cells in MAlignant Tumor tissues using Expression data (ESTIMATE) algorithm using “estimate” R package^15^. The correlation of TEDC2 expression with the abundance of six types of infiltrating immune cells (CD8+ T cells, CD4+ T cells, macrophages, B cells, dendritic cells (DCs) and neutrophils) in LUAD was evaluated by TIMER2.0 database (http://timer.cistrome.org/)^16^.

To study the relationship between TEDC2 gene expression and immune checkpoints in LUAD, we extracted 47 common immune checkpoints and used Spearman’s rank correlation coefficient to analyze the correlation. The associations of TEDC2 expression with multiple markers for immune cells and immune checkpoints leading to T cell exhaustion were further investigated in the Gene Expression Profiling Interactive Analysis (GEPIA) 2 database which is an online database providing key interactive and customizable functions^17^.

### Statistical analysis

The patients were divided into two groups according to the median expression of TEDC2. The measurement data were statistically analyzed with t test if normally distributed. Otherwise, Mann–Whitney U test was used. The receiver operating characteristic (ROC) curve was used to evaluate the potential diagnostic value of TEDC2. Overall survival (OS), disease specific survival (DSS) and progress free survival (PFS) analyses were performed by Kaplan–Meier curve and log-rank test. Univariate and multivariable analyses were conducted by the Cox proportional hazards regression models to determine the prognostic value of TEDC2 in survival outcomes. Multivariate regression model included the factors that *P* value was less than 0.1 in univariate analysis. R statistical software (version 3.6.3) and SPSS software (version 24.0) were used to perform the analyses in the study. Statistical significance was set at *P* value < 0.05 (All *P* values presented were 2-sided).

## Results

### TEDC2 is upregulated in LUAD

**Figure 1:**
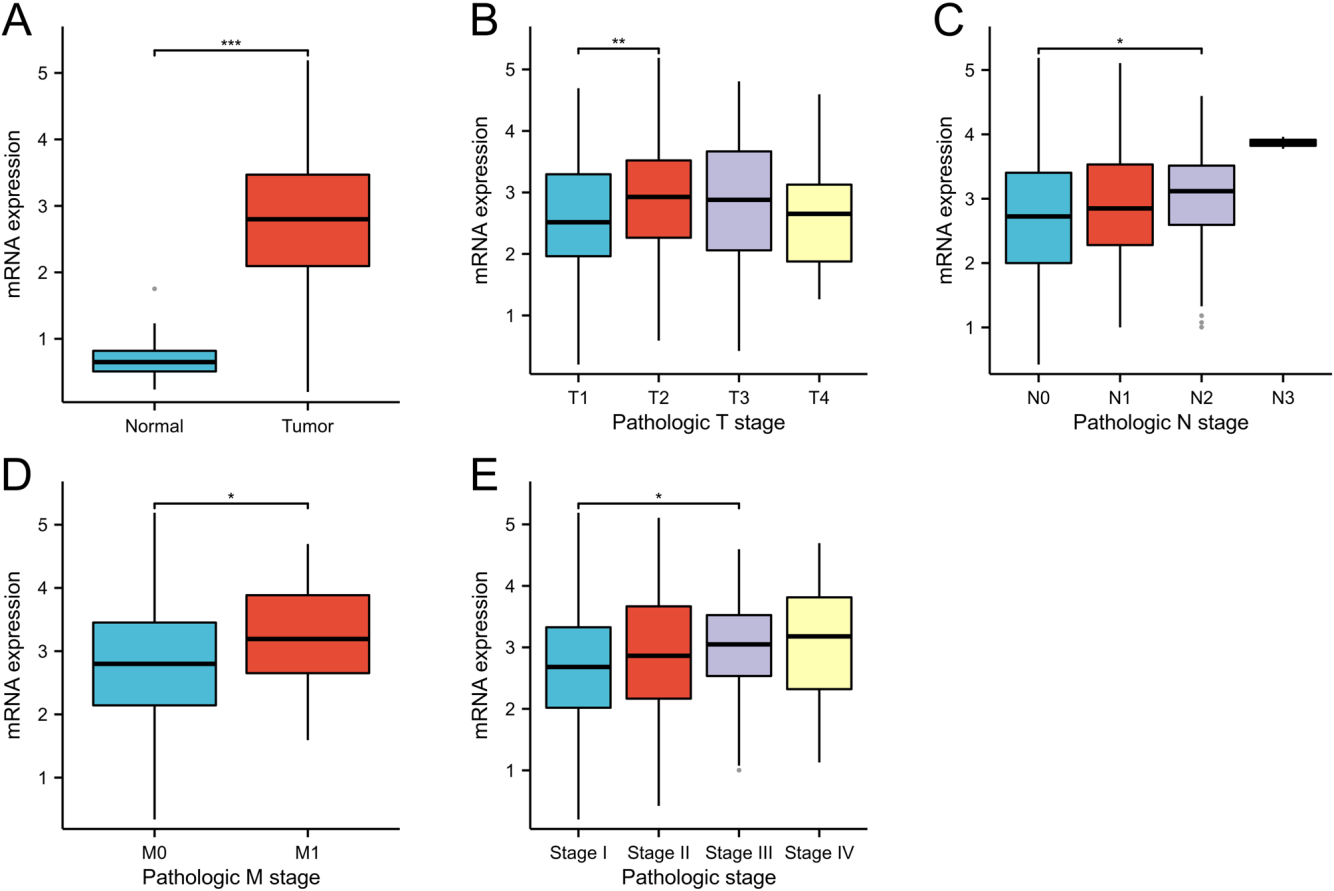
TEDC2 expression status in LUAD. (**A**) TEDC2 expression was higher in LUAD tissues than in normal tissues. (**B**) High TEDC2 expression was associated with advanced T stage. (**C**) High TEDC2 expression was associated with advanced N stage. (**D**) High TEDC2 expression was associated with advanced M stage. (**E**) High TEDC2 expression was associated with advanced pathologic stage. *P < 0.05; **P < 0.01; ***P < 0.001. The figure was created by R statistical software (version 3.6.3).

The baseline characteristics of patients with LUAD obtained from TCGA database were summarized in Table 1. The expression of TEDC2 in LUAD samples was significantly upregulated at mRNA levels in TCGA database (*P* < 0.001, Fig. 1A), and the mRNA expression of TEDC2 was positively correlated with advanced T stage (T1 vs. T2, *P* = 0.008, Fig. 1B), N stage (N0 vs. N2, *P* = 0.011, Fig. 1C), M stage (M0 vs. M1, *P* = 0.044, Fig. 1D) and pathologic stage (stage I vs. stage III, *P* = 0.025, Fig. 1E), but the significant differences were not observed in T3 and T4, N1 and N3, stage II and stage IV.

**Table 1:** Baseline characteristics of the LUAD patients.

Characteristics	N	(%)
Age		
> 65	261	50.6
≤ 65	255	49.4
Gender		
Male	249	46.5
Female	286	53.5
Smoking history		
Yes	446	85.6
No	75	14.4
T stage		
T1	175	32.9
T2	289	54.3
T3	49	9.2
T4	19	3.6
N stage		
N0	348	67.1
N1	95	18.3
N2	74	14.3
N3	2	0.4
M stage		
M0	361	93.5
M1	25	6.5
Pathologic stage		
Stage I	294	55.8
Stage II	123	23.3
Stage III	84	15.9
Stage IV	26	4.9
TEDC2 expression		
High	268	50.1
Low	267	49.9

**Figure 2:**
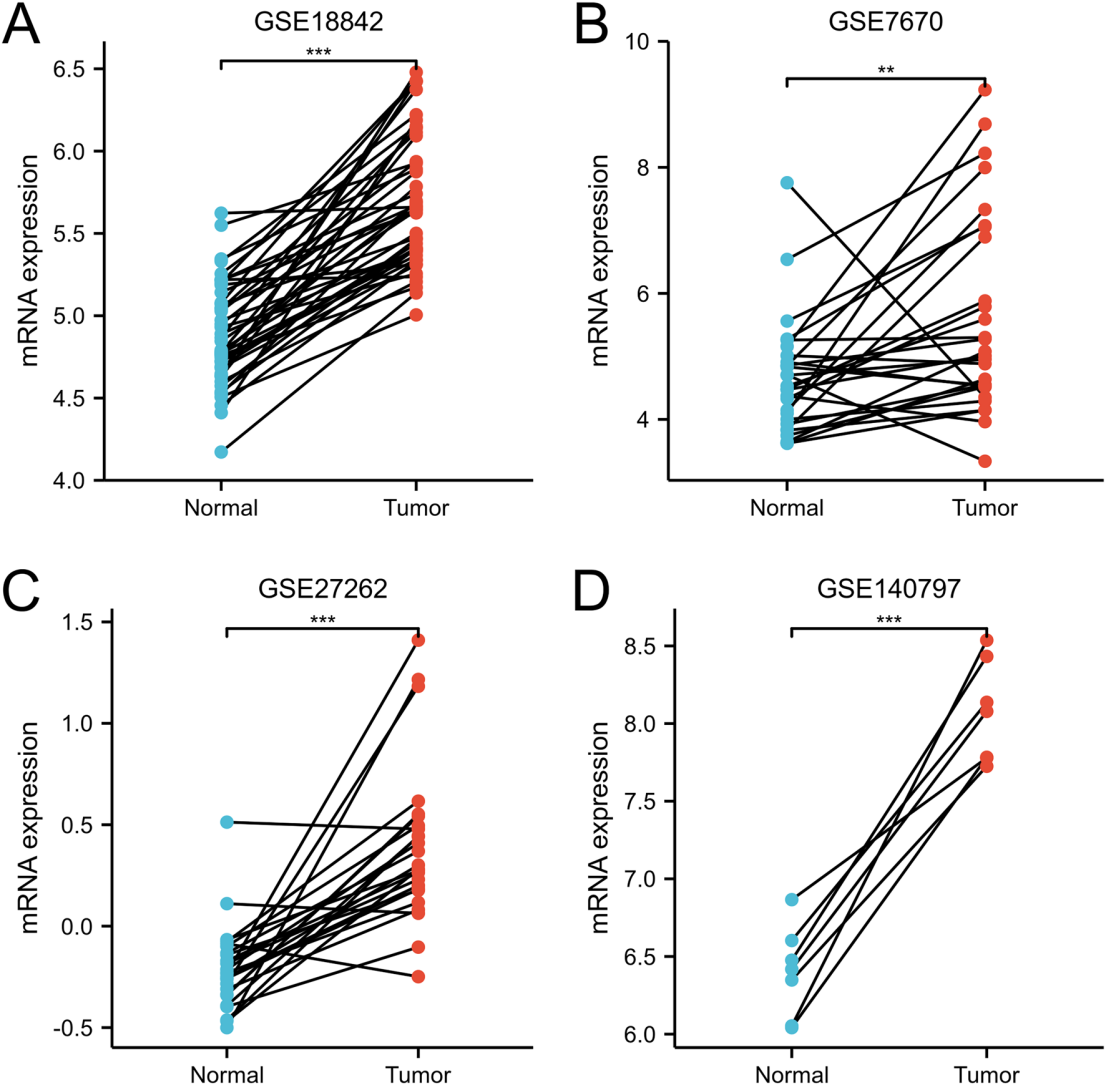
Validation of higher TEDC2 expression in LUAD than that in normal tissues in (**A**) GSE18842, (**B**) GSE7670, (**C**) GSE27262 and (**D**) GSE140797 datasets. **P < 0.01; ***P < 0.001. The figure was created by R statistical software (version 3.6.3).

**Figure 3:**
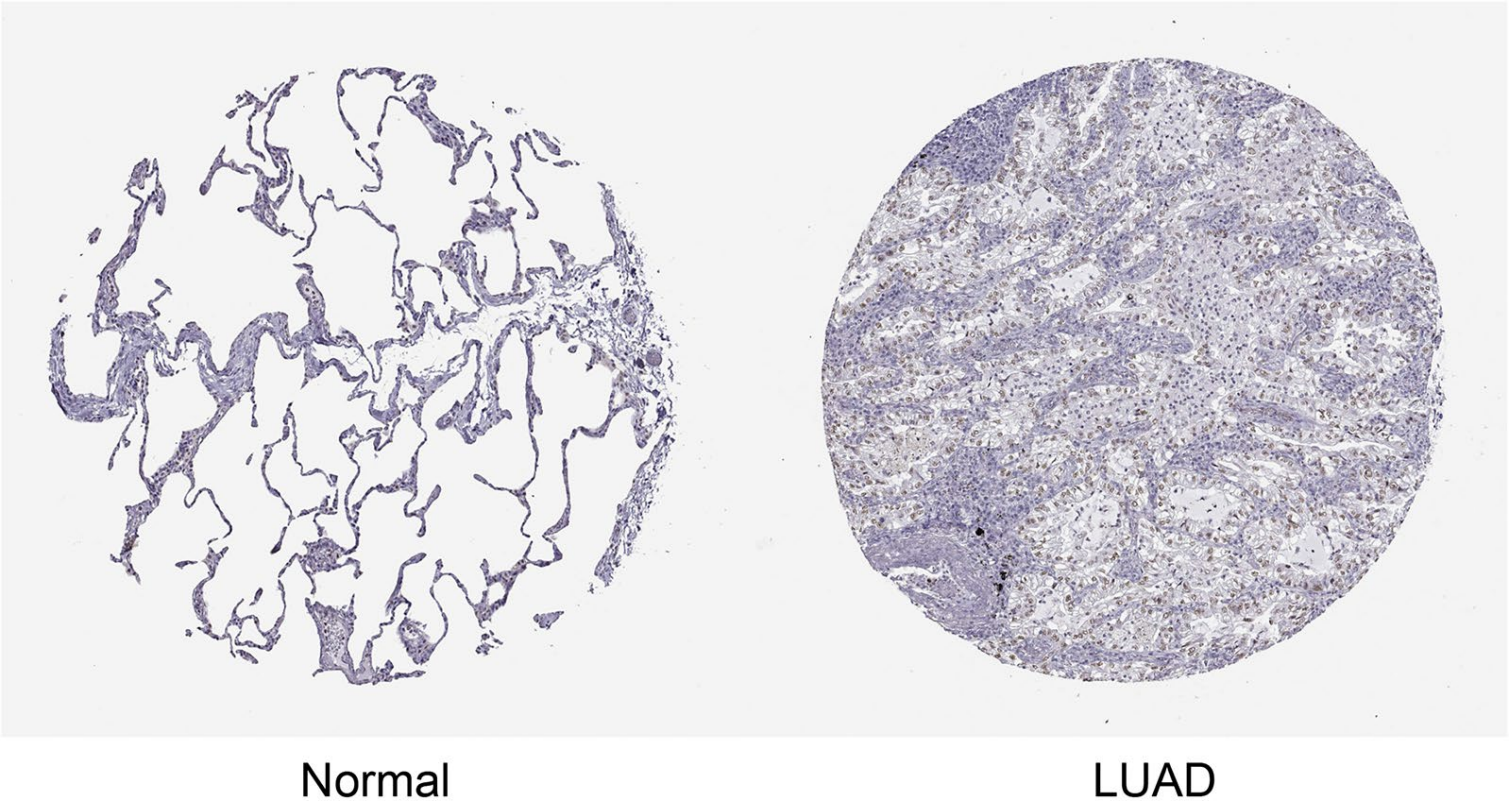
The protein level of TEDC2 was higher in LUAD tissue than normal tissue in the Human Protein Atlas (Antibody HPA055389, 10X). The figure was download in Human Protein Atlas (https://www.proteinatlas.org/).

The differential expression of TEDC2 between LUAD and normal tissues was verified by the data sets GSE18842 (44 pairs LUAD and adjacent normal tissues), GSE7670 (28 pairs LUAD and adjacent normal tissues), GSE27262 (25 pairs LUAD and adjacent normal tissues) and GSE140797 (7 pairs LUAD and adjacent normal tissues). The results confirmed that mRNA expression of TEDC2 was higher in LUAD tissues than that in paracancer tissues (Fig. 2A–D). The expression of TEDC2 at the protein level was also analyzed in the Human Protein Atlas, and we found that TEDC2 protein expression increased significantly in patients with LUAD (Fig. 3).

### TEDC2 possesses diagnostic and prognostic value for LUAD

**Figure 4:**
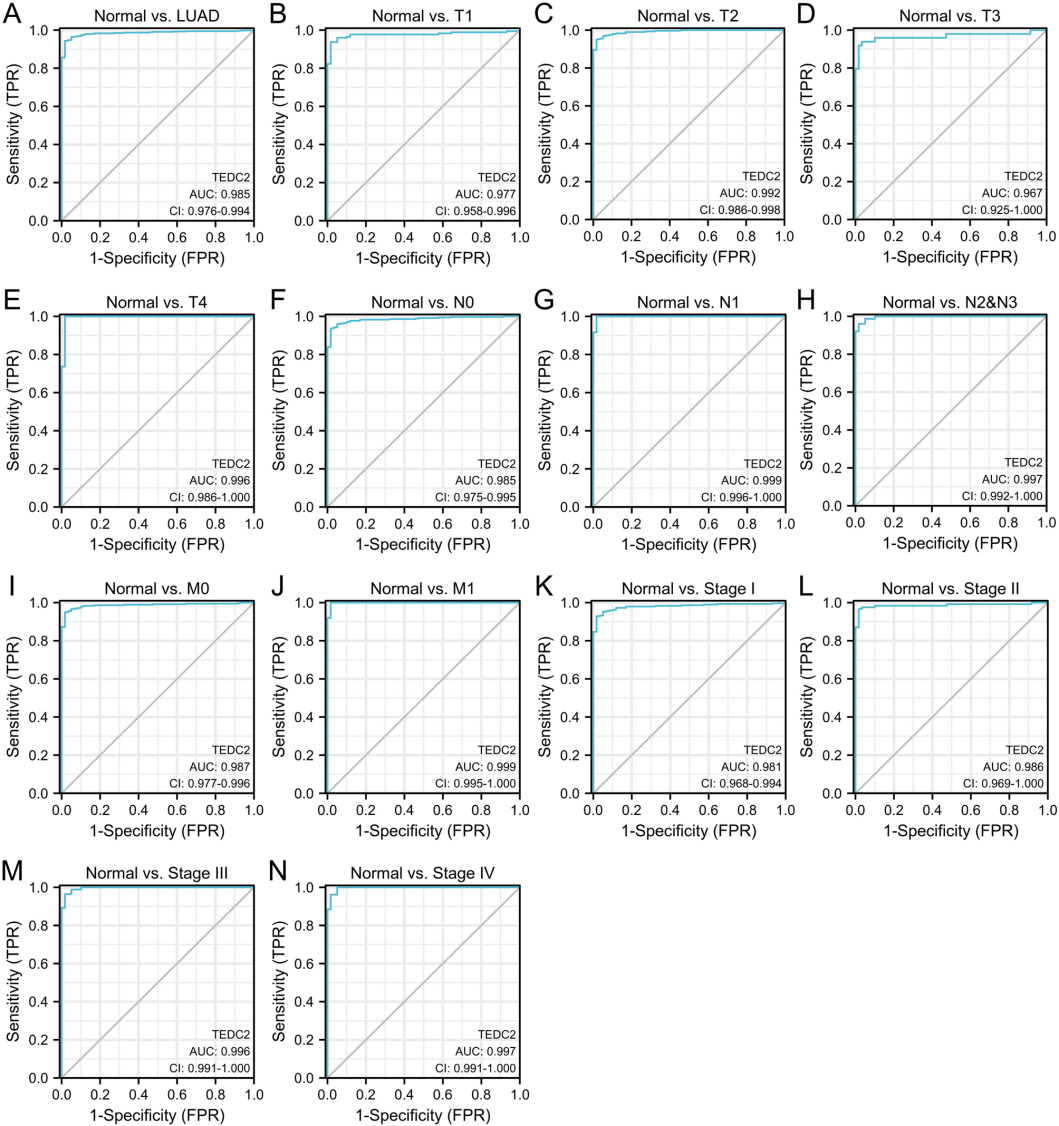
Analysis of the diagnostic value of TEDC2. (**A**) ROC curve analysis of the diagnostic value of TEDC2 in patients with LUAD. (**B**–**E**) ROC curve analysis of the diagnostic value of TEDC2 in patients with LUAD at T stage. (**F**–**H**) ROC curve analysis of the diagnostic value of TEDC2 in patients with LUAD at N stage. (**I**,**J**) ROC curve analysis of the diagnostic value of TEDC2 in patients with LUAD at M stage. (**K**–**N**) ROC curve analysis of the diagnostic value of TEDC2 in patients with LUAD at pathologic stage. The figure was created by R statistical software (version 3.6.3).

The diagnostic value of TEDC2 was determined by ROC curve analyses which were performed between tumor tissues with different stage and normal tissues. As shown in Fig. 4A, high TEDC2 levels could effectively distinguish LUAD tissues from normal tissues. Then, subtype analyses were performed in different TNM stage, and the results suggested that TEDC2 possessed satisfactory diagnostic value regardless of early or advanced stage (Fig. 4B–N).

**Figure 5:**
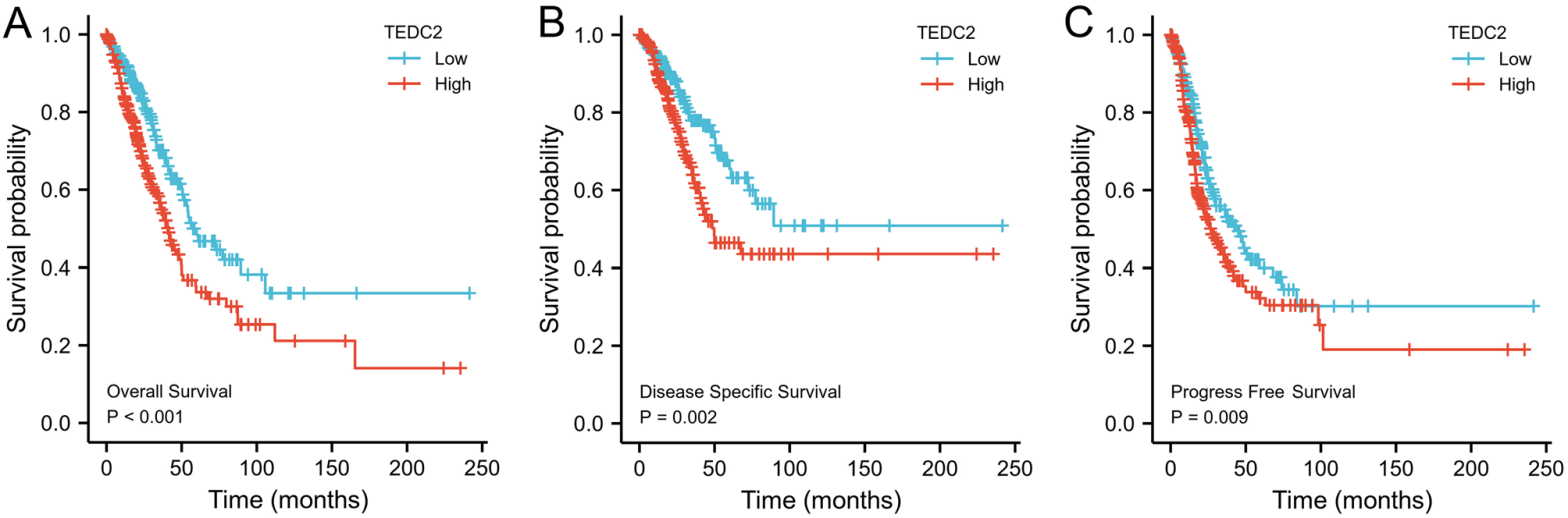
Kaplan–Meier survival curves for (**A**) overall survival (OS), (**B**) disease specific survival (DSS) and (**C**) progress free survival (PFS) of the LUAD patients with high and low TEDC2 expression level. The figure was created by R statistical software (version 3.6.3).

**Figure 6:**
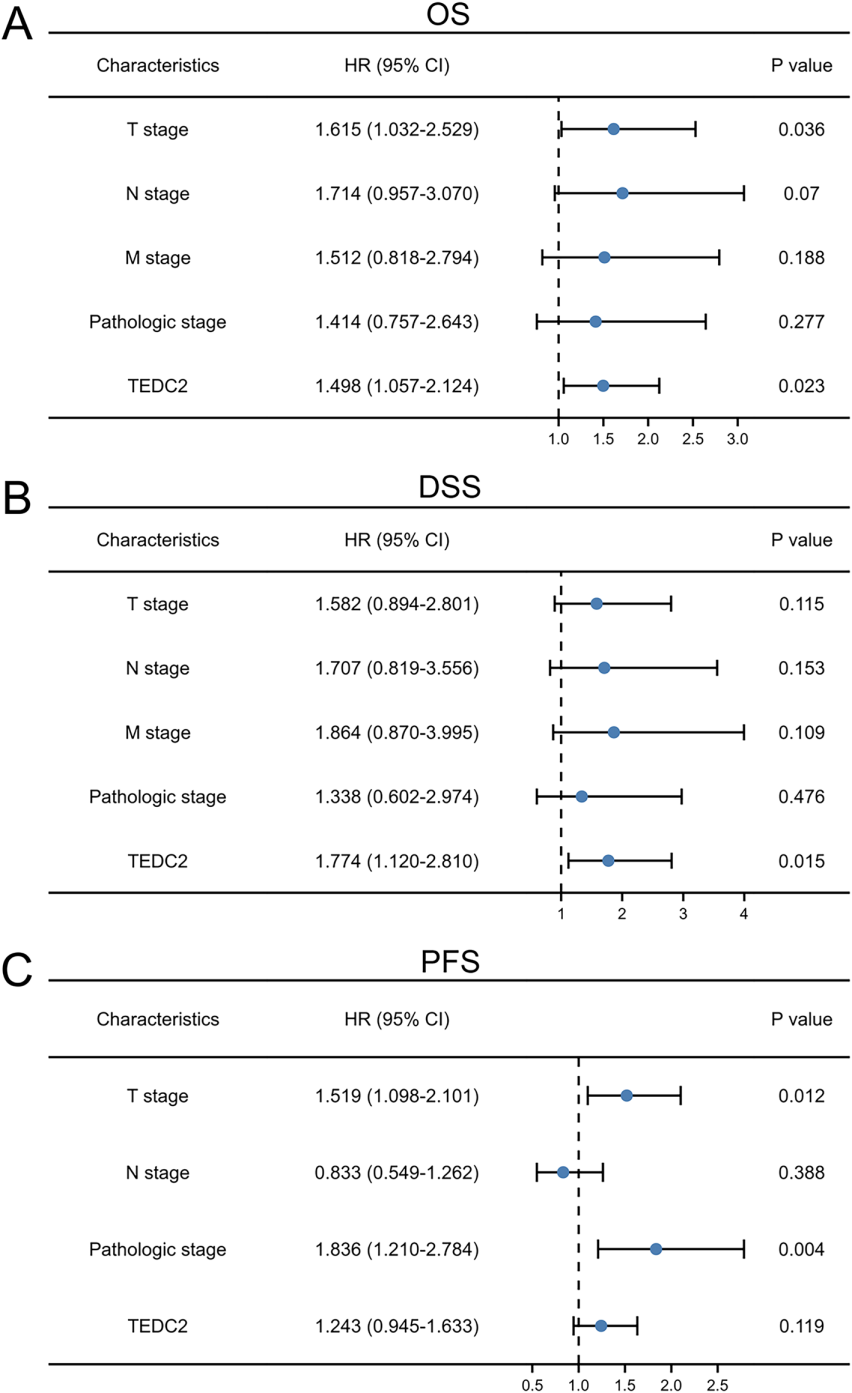
Multivariate Cox regression analysis of TEDC2 expression and clinicopathologic characteristics with survival outcomes in LUAD. (**A**) Multivariate Cox regression analysis in overall survival (OS). (**B**) Multivariate Cox regression analysis in disease specific survival (DSS). (**C**) Multivariate Cox regression analysis in progress free survival (PFS). The figure was created by R statistical software (version 3.6.3).

Kaplan–Meier analysis suggested that high expression of TEDC2 was significantly associated with poor OS (*P* < 0.001, Fig. 5A), DSS (*P* = 0.002, Fig. 5B) and PFS (*P* = 0.009, Fig. 5C) in LUAD patients. Univariate Cox regression analysis indicated that TEDC2 expression was correlated with OS (HR 1.704, 95% CI 1.273–2.282), DSS (HR 1.796, 95% CI 1.240–2.601) and PFS (HR 1.422, 95% CI 1.092–1.851) (Table 2). Furthermore, multivariate Cox regression revealed that TEDC2 expression was an independent adverse prognostic indicator for OS (HR 1.498, 95% CI 1.057–2.124, *P* = 0.023) and DSS (HR 1.774, 95% CI 1.120–2.810, *P* = 0.015) in LUAD patients (Fig. 6).

**Table 2:** Cox regression analysis of TEDC2 and clinicopathologic characteristics with survival outcomes in LUAD.

Characteristics	Univariate analysis			Multivariate analysis		
	HR	95% CI	*P*	HR	95% CI	*P*
Overall survival						
Age (> 65 vs. ≤ 65)	1.223	0.916–1.635	0.172			
Gender (Male vs. Female)	1.070	0.803–1.426	0.642			
Smoking history (Yes vs. No)	0.894	0.592–1.348	0.591			
T stage (T2 & T3 & T4 vs. T1)	1.728	1.229–2.431	0.002	1.615	1.032–2.529	0.036
N stage (N1 & N2 & N3 vs. N0)	2.601	1.944–3.480	< 0.001	1.714	0.957–3.070	0.070
M stage (M1 vs. M0)	2.136	1.248–3.653	0.006	1.512	0.818–2.794	0.188
Pathologic stage (Stage II & Stage III & Stage IV vs. Stage I)	2.933	2.173–3.958	< 0.001	1.414	0.757–2.643	0.277
TEDC2 (High vs. Low)	1.704	1.273–2.282	< 0.001	1.498	1.057–2.124	0.023
Disease specific survival						
Age (> 65 vs. ≤ 65)	1.013	0.701–1.464	0.944			
Gender (Male vs. Female)	0.989	0.687–1.424	0.954			
Smoking history (Yes vs. No)	1.040	0.602–1.796	0.889			
T stage (T2 & T3 & T4 vs. T1)	1.850	1.195–2.865	0.006	1.582	0.894–2.801	0.115
N stage (N1 & N2 & N3 vs. N0)	2.703	1.873–3.900	< 0.001	1.707	0.819–3.556	0.153
M stage (M1 vs. M0)	2.455	1.269–4.749	0.008	1.864	0.870–3.995	0.109
Pathologic stage (Stage II & Stage III & Stage IV vs. Stage I)	3.291	2.237–4.842	< 0.001	1.338	0.602–2.974	0.476
TEDC2 (High vs. Low)	1.796	1.240–2.601	0.002	1.774	1.120–2.810	0.015
Progress free survival						
Age (> 65 vs. ≤ 65)	1.023	0.784–1.335	0.867			
Gender (Male vs. Female)	1.172	0.901–1.526	0.236			
Smoking history (Yes vs. No)	0.968	0.658–1.426	0.870			
T stage (T2 & T3 & T4 vs. T1)	1.882	1.379–2.570	< 0.001	1.519	1.098–2.101	0.012
N stage (N1 & N2 & N3 vs. N0)	1.512	1.152–1.984	0.003	0.833	0.549–1.262	0.388
M stage (M1 vs. M0)	1.513	0.855–2.676	0.155			
Pathologic stage (Stage II & Stage III & Stage IV vs. Stage I)	1.960	1.502–2.557	< 0.001	1.836	1.210–2.784	0.004
TEDC2 (High vs. Low)	1.422	1.092–1.851	0.009	1.243	0.945–1.633	0.119

### TEDC2 co-expression network in LUAD

**Figure 7:**
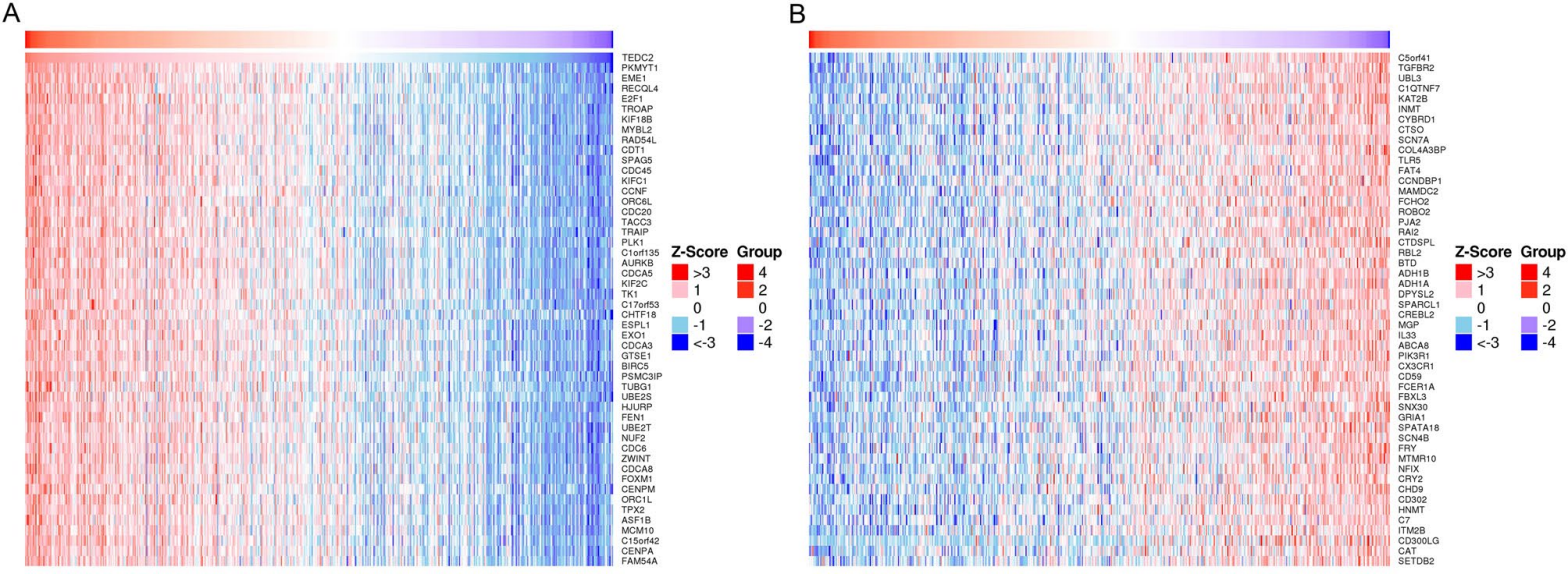
The co-expression genes with TEDC2 in LUAD from the LinkedOmics database. (**A**,**B**) Heat maps of top 50 genes positively and negatively correlated to TEDC2 in LUAD, respectively. The figure was created by the LinkedOmics database (http://www.linkedomics.org).

**Figure 8:**
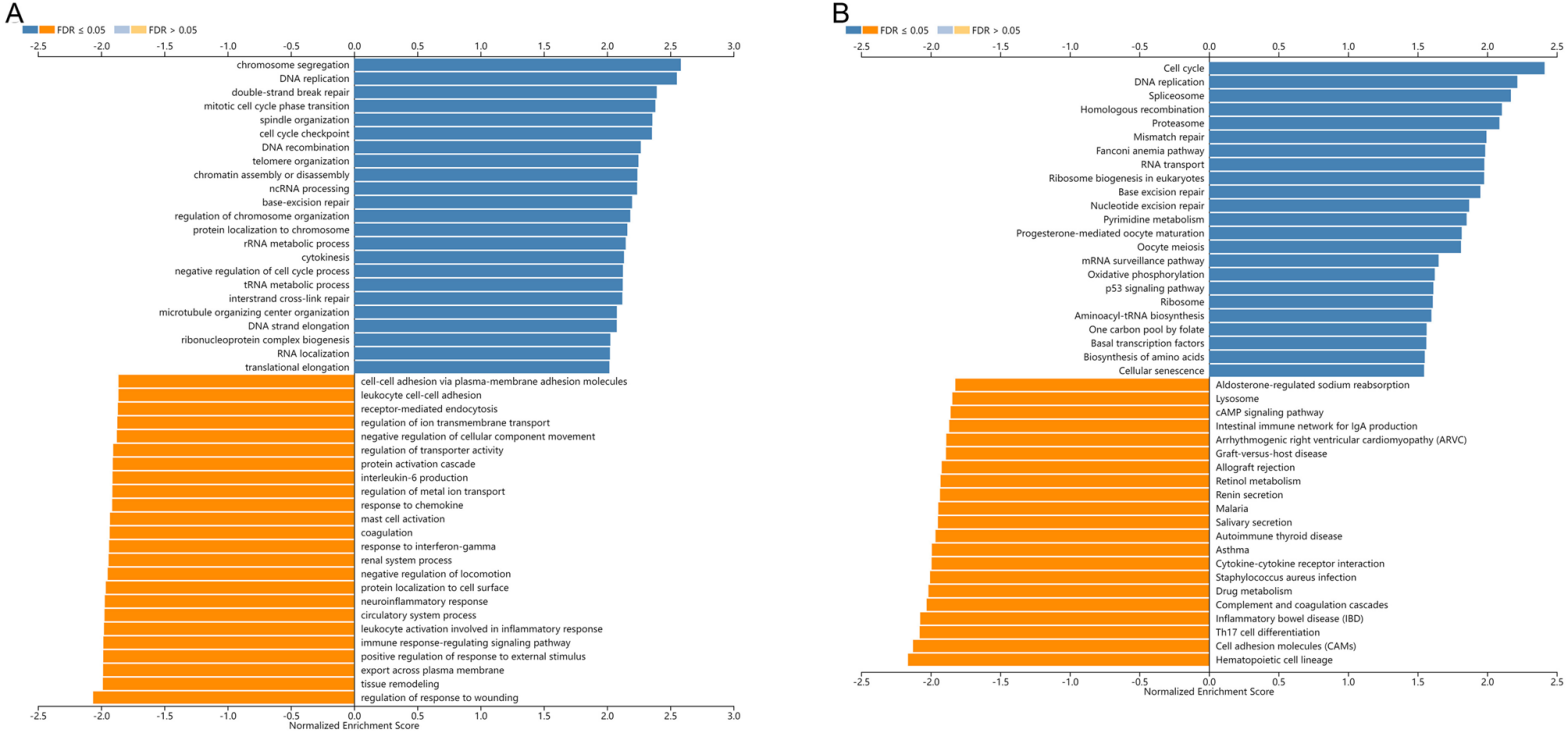
(**A**) GO annotations and (**B**) KEGG pathways of TEDC2 in LUAD (Kanehisa, M. and Goto, S., 2020. KEGG: Kyoto Encyclopedia of Genes and Genomes. Nucleic Acids Res. 28, 27-30). The figure was created by the LinkedOmics database (http://www.linkedomics.org).

The LinkFinder module in the LinkedOmics database was used to explore the co-expression pattern of TEDC2 in LUAD. The results showed that 10,003 genes were positively correlated with TEDC2, while 9985 genes were negatively correlated with TEDC2 (Supplementary figure 1). Heat maps displayed the top 50 genes positively and negatively associated with TEDC2 (Fig. 7A,B), and correlation coefficients and *P* values were presented in supplementary table 1 and 2. In the LinkInterpreter module, GO term annotation showed that co-expressed genes of TEDC2 were mainly involved in chromosome segregation, DNA replication, double-strand break repair, mitotic cell cycle phase transition, spindle organization, cell cycle checkpoint, DNA recombination, telomere organization, chromatin assembly or disassembly, ncRNA processing, etc. (Fig. 8A). KEGG pathway analysis indicated enrichment in cell cycle, DNA replication, spliceosome, homologous recombination, proteasome, mismatch repair, Fanconi anemia pathway, RNA transport, ribosome biogenesis in eukaryotes, base excision repair, etc. (Fig. 8B).

### TEDC2 is correlated with immune infiltration in LUAD

**Figure 9:**
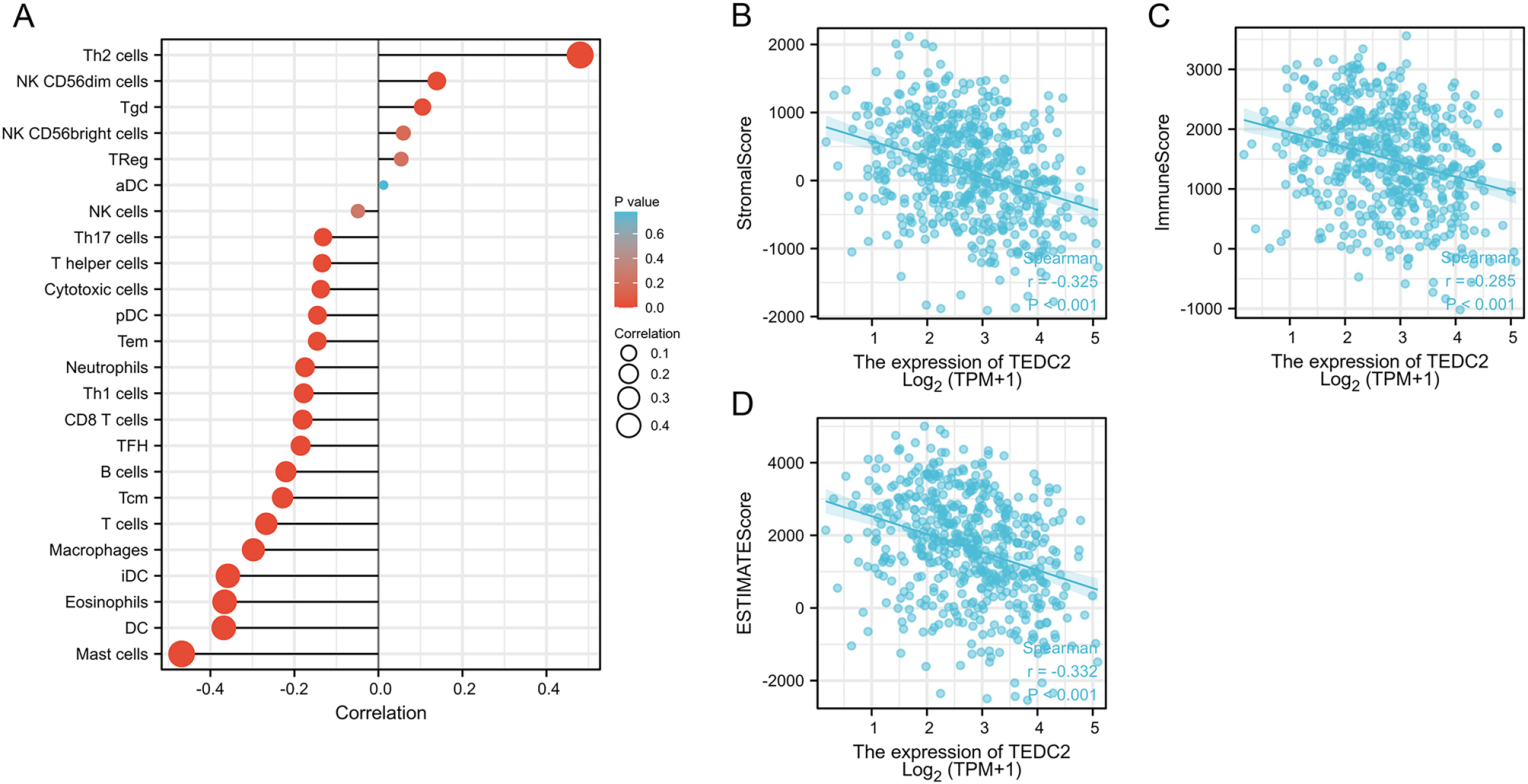
The correlation between TEDC2 expression and immune microenvironment in LUAD. (**A**) The correlation of TEDC2 expression with infiltrated immune cells by ssGSEA algorithm. (**B**–**D**) The correlation of TEDC2 expression with stromal score, immune score, and ESTIMATE score by ESTIMATE algorithm, respectively. The figure was created by R statistical software (version 3.6.3).

**Figure 10:**
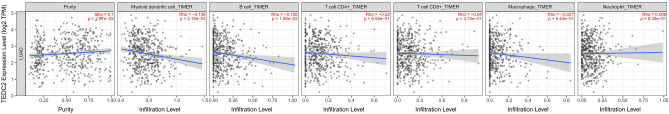
The correlation of TEDC2 expression with immune infiltration in LUAD from the TIMER2.0 database. The figure was created by TIMER2.0 database (http://timer.cistrome.org/).

The ssGSEA and the ESTIMATE algorithms were employed to characterize the immune features of TEDC2. According to the ssGSEA algorithm, the expression of TEDC2 was negatively correlated with most infiltrated immune cells (Fig. 9A). Moreover, the expression of TEDC2 was significantly negatively associated with the stromal score of LUAD (R = − 0.325, *P* < 0.001, Figs. 9B), as well as the immune score (R = − 0.285, *P* < 0.001, Fig. 9C). The ESTIMATE score which was calculated as the sum of the stromal and immune scores was also negatively correlated with the expression of TEDC2 (R = − 0.332, *P* < 0.001, Figs. 9D). The correlation of TEDC2 expression with immune cell infiltration was further explored in the TIMER2.0 database. The result indicated significantly negative association of TEDC2 expression with DC (R = − 0.138, *P* = 2.18e−03) and B cell (R = − 0.108, *P* = 1.65e−02) (Fig. 10).

**Figure 11:**
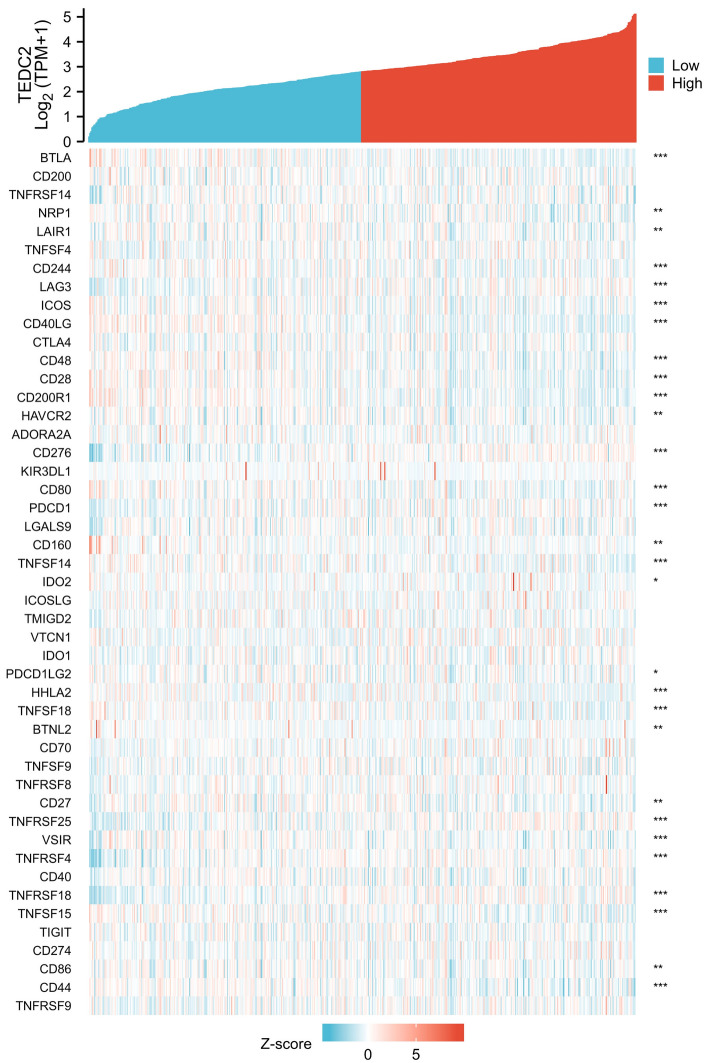
The correlation of TEDC2 expression with common immune checkpoints. The figure was created by R statistical software (version 3.6.3).

The analysis of the relationship between TEDC2 expression and immune checkpoints showed that TEDC2 was associated with most immune checkpoints in LUAD (Fig. 11), among which TEDC2 was significantly positively correlated with lymphocyte activating 3 (LAG3), CD276, programmed cell death 1 (PDCD1), tumor necrosis factor receptor superfamily member (TNFRSF) 25, TNFRSF4 and TNFRSF18 (Table 3). To further investigate the impact of TEDC2 on immune microenvironment in LUAD, the associations of TEDC2 with immune marker sets of immune cells and immune checkpoints leading to T cell exhaustion were analyzed in the GEPIA2 database (Table 4). We found that TEDC2 expression was negatively associated with the levels of some marker sets marking DC, B cell, tumor-associated macrophage (TAM), monocyte and neutrophil. Moreover, TEDC2 expression was positively correlated with immune checkpoints leading to T cell exhaustion such as PDCD1, LAG3 and CD276.

**Table 3:** The relationship between TEDC2 and immune checkpoints.

Immune checkpoints	R	*P*
LAG3	0.243	< 0.001
CD276	0.359	< 0.001
PDCD1	0.164	< 0.001
TNFRSF25	0.324	< 0.001
TNFRSF4	0.194	< 0.001
TNFRSF18	0.314	< 0.001

**Table 4:** Correlation analysis between TEDC2 and markers of immune cells in GEPIA2 database.

Cell type	Gene marker	Normal		Tumor	
		R	*P*	R	*P*
B cell	CD19	0.27	0.042	− 0.081	0.074
	CD79A	0.055	0.68	− 0.17	0.00013
CD8+ T cell	CD8A	− 0.013	0.92	− 0.036	0.43
	CD8B	0.034	0.8	− 0.005	0.91
Tfh	BCL6	− 0.41	0.0014	− 0.003	0.95
	IL21	0.19	0.15	0.065	0.15
Th1	TBX21	0.14	0.28	− 0.039	0.39
	STAT4	− 0.017	0.9	− 0.012	0.79
	STAT1	0.2	0.13	0.22	1e−06
	IFNG	0.24	0.071	0.11	0.021
	TNF	0.3	0.022	− 0.057	0.21
Th2	GATA3	0.21	0.11	− 0.01	0.82
	IL13	− 0.15	0.27	0.0043	0.93
	STAT6	0.17	0.2	− 0.049	0.28
	STAT5A	0.34	0.0081	− 0.086	0.059
Th17	STAT3	− 0.26	0.046	0.043	0.35
	IL17A	− 0.17	0.2	− 0.019	0.68
Treg	FOXP3	0.42	0.00097	0.01	0.82
	STAT5B	− 0.056	0.67	0.046	0.32
	CCR8	0.23	0.086	− 0.077	0.092
	TGFB1	0.27	0.037	− 0.066	0.15
M1	NOS2	− 0.018	0.89	0.068	0.13
	IRF5	0.47	0.00019	0.063	0.17
	PTGS2	− 0.32	0.015	0.098	0.032
M2	CD163	0.066	0.62	− 0.062	0.17
	VSIG4	0.041	0.76	− 0.19	3e−05
	MS4A4A	0.083	0.53	− 0.24	1.1e−07
TAM	CCL2	− 0.25	0.055	− 0.031	0.49
	CD68	0.11	0.4	− 0.12	0.0078
	IL10	0.1	0.44	− 0.12	0.0078
Monocyte	CD86	0.069	0.6	− 0.16	0.00032
	CD115	0.44	0.00046	− 0.14	0.0028
Neutrophil	CD66b	0.093	0.48	− 0.28	1.9e−10
	CCR7	0.37	0.0035	− 0.21	3.1e−06
	CD11b	0.17	0.21	− 0.14	0.0017
Natural killer cell	XCL1	0.0023	0.99	0.065	0.15
	CD7	0.26	0.048	0.2	8.3e−06
	KIR3DL1	− 0.022	0.87	0.033	0.46
Dendritic cell	CD1C	0.17	0.19	− 0.44	1.5e−24
	CD141	− 0.28	0.034	− 0.25	3.3e−08
	CD11c	0.024	0.86	0.045	0.33
T cell exhaustion	PDCD1	0.14	0.28	0.13	0.0054
	CTLA4	0.057	0.67	0.021	0.65
	LAG3	0.31	0.019	0.19	1.7e−05
	CD276	0.19	0.14	0.31	1.4e−12

Tfh, follicular helper T cell; Th, T helper cell; Treg, regulatory T cell; TAM, tumor-associated macrophage.

## Discussion

The past decade in the LUAD research has been characterized by a greater understanding of cancer biology and management, with targeted therapy and immunotherapy providing significant survival benefits and manageable toxicity profiles in selected patients. However, major challenges still remain, including low response rate and drug resistance^18,19^. Thus, there is a clear urgent need to identify new driver gene alterations to expand the population that benefit from targeted therapy or immunotherapy, predict treatment responses and prevent or overcome the drug resistance. In this study, through employing open-access databases for comprehensive analyses, we found that TEDC2 could be involved in the tumorigenesis and progression of LUAD, and might contribute to the formation of immunosuppressive microenvironment in LUAD patients. These results suggested that TEDC2 could be regarded as a novel potential target for the treatment of LUAD.

The role of TEDC2 has been explored in few studies. Lim, D. H. et al. reported that TEDC2 could be predominantly expressed in primary central nervous system (CNS) diffuse large B-cell lymphoma (DLBCL) compared to non-CNS DLBCL^11^. Hsu, M. K. et al. suggested that TEDC2 might be one of the potential genes for the tumorigenesis of LUAD and the construction of accurate classification systems distinguishing tumor from normal tissues^10^. TEDC2 was also seemed to act as a potential marker for treatment effect in male schizophrenia patients^20^. However, TEDC2 is not yet thoroughly studied and currently its function remains unclear. Our study preliminarily demonstrated a part of functions of TEDC2 in LUAD.

According to the analyses of data from TCGA and GEO databases, the mRNA expression of TEDC2 was significantly upregulated in LUAD compared to normal tissues. It was worth mentioning that although the sample of GSE140797 was relatively small, significant differences of TEDC2 expression were detected in all pairs. Moreover, we have used other datasets from GEO database to confirm the result of the GSE140797 analysis. The protein level of TEDC2 was also confirmed to be higher in LUAD by immunohistochemistry analysis in Human Protein Atlas. In addition, ROC curve analysis found that TEDC2 could distinguish patients with LUAD from the normal population regardless of tumor stage. These evidences indicated that TEDC2 might play an important role in the tumorigenesis of LUAD and could serve as a new diagnostic marker for LUAD patients. To determine whether TEDC2 could be used as a prognostic marker in LUAD, we investigated the prognosis of LUAD patients with different TEDC2 expression levels. Kaplan–Meier curve analysis revealed that high TEDC2 expression was associated with inferior survival outcomes including OS, DSS and PFS. Cox regression analysis proved that high TEDC2 expression was an independent risk factor for poor prognosis, suggesting the potential prognostic value of TEDC2 in LUAD.

To unravel the biological functions of TEDC2, co-expression analysis and functional enrichment analysis were performed. LinkedOmics database analysis pointed out that most co-expressed genes with TEDC2 were mainly enriched in mitotic cell cycle processes, including chromosome segregation, DNA replication and cell cycle phase transition, suggesting that these genes could act as oncogenes to promote LUAD by accelerating cell cycle phase.

Another vital aspect of this study was that TEDC2 might be involved in regulating immune microenvironment in LUAD. There is increasing evidence proving the important role of tumor immune microenvironment in cancers^21,22^. With the application of ssGSEA and ESTIMATE algorithms, we identified that the expression of TEDC2 was significantly negatively associated with immune infiltrates, which implied that TEDC2 might induce immunosuppressive context. The relationships between TEDC2 and immune infiltrates in LUAD were also analyzed by TIMER2.0 and GEPIA2 databases. The results demonstrated that TEDC2 expression showed negative correlation with DC and B cell. DC is one of the major regulators of immune response and can elicit T cell response, and previous studies have proved that DC could be associated with cytotoxic T cell infiltration and predict favorable outcome^23,24^. B cell has emerged as a key player in immune microenvironment and correlates with better prognosis in NSCLC^25,26^.

In addition, we conducted a systematic analysis of more than 40 common immune checkpoint genes and found that PDCD1, LAG3 and CD276 were highly correlated with TEDC2 expression, and the analysis of GEPIA2 database also proved these correlations. PDCD1 is an inhibitory receptor and negative regulator of T cell function, which can promote disease progression in patients with NSCLC^27^. LAG3 is able to function in coordination with other checkpoints such as PDCD1 to inhibit the activity of effector T cells and promote the suppressive activity, but effects and signaling events after LAG3 activation have not been completely understood^28^. CD276 expression on lung cancer leads to a lower number of tumor infiltrating lymphocytes and promotes lymph node metastasis, suggesting a role for CD276 in immune evasion and tumor progression^29^. Besides, TEDC2 expression was also correlated with TNFRSF25, TNFRSF4 and TNFRSF18 in our analysis, which might be correlated with immune evasion and poor outcome in lung cancer, but the molecular mechanisms of these immune checkpoint molecules still remain elusive and need further investigation^30,31^.

This study preliminarily demonstrated the diagnostic and prognostic values of TEDC2 in LUAD, as well as the immune characteristics. However, there were certain limitations in our study. First, data heterogeneity was inevitable due to all the data in this study obtained from online databases. Second, gene expression analysis based on open-source databases might not be sufficiently accurate and the experiment examining biological functions of TEDC2 lacked, which required additional experiments to provide a better understanding of the underlying biological mechanisms of TEDC2. Finally, although the regulatory effect of TEDC2 on immune microenvironment was evaluated by various algorithms in this study and the results reached statistical significance, the actual status of specific immune processes should be further investigated by in vitro and vivo models.

## Conclusion

The preliminary results in this study characterize the clinical and immune features of TEDC2 in LUAD. The high expression of TEDC2 was associated with poor prognosis and immunosuppressive microenvironment. TEDC2 could be used as a biomarker to predict the prognosis and as a potential target for treatment in LUAD patients.

## Supplementary Information

Supplementary Figure S1.

Supplementary Tables.

## Data Availability

The following information was supplied regarding data availability: Data is available at NCBI GEO: GSE18842, GSE7670, GSE27262 and GSE140797 (https://www.ncbi.nlm.nih.gov/gds). The expression profile and clinical data are available at the TCGA database (https://portal.gdc.cancer.gov/) (level 3 HTseq-FPKM format from the LUAD (Lung Adenocarcinoma) project).
