# Supplementary figures and images for: TEDC2 correlated with prognosis and immune microenvironment in lung adenocarcinoma
Source: Sci Rep. 2023 Mar 27;13:5006. doi: 10.1038/s41598-023-32238-8 (PMC10042817; doi:10.1038/s41598-023-32238-8)

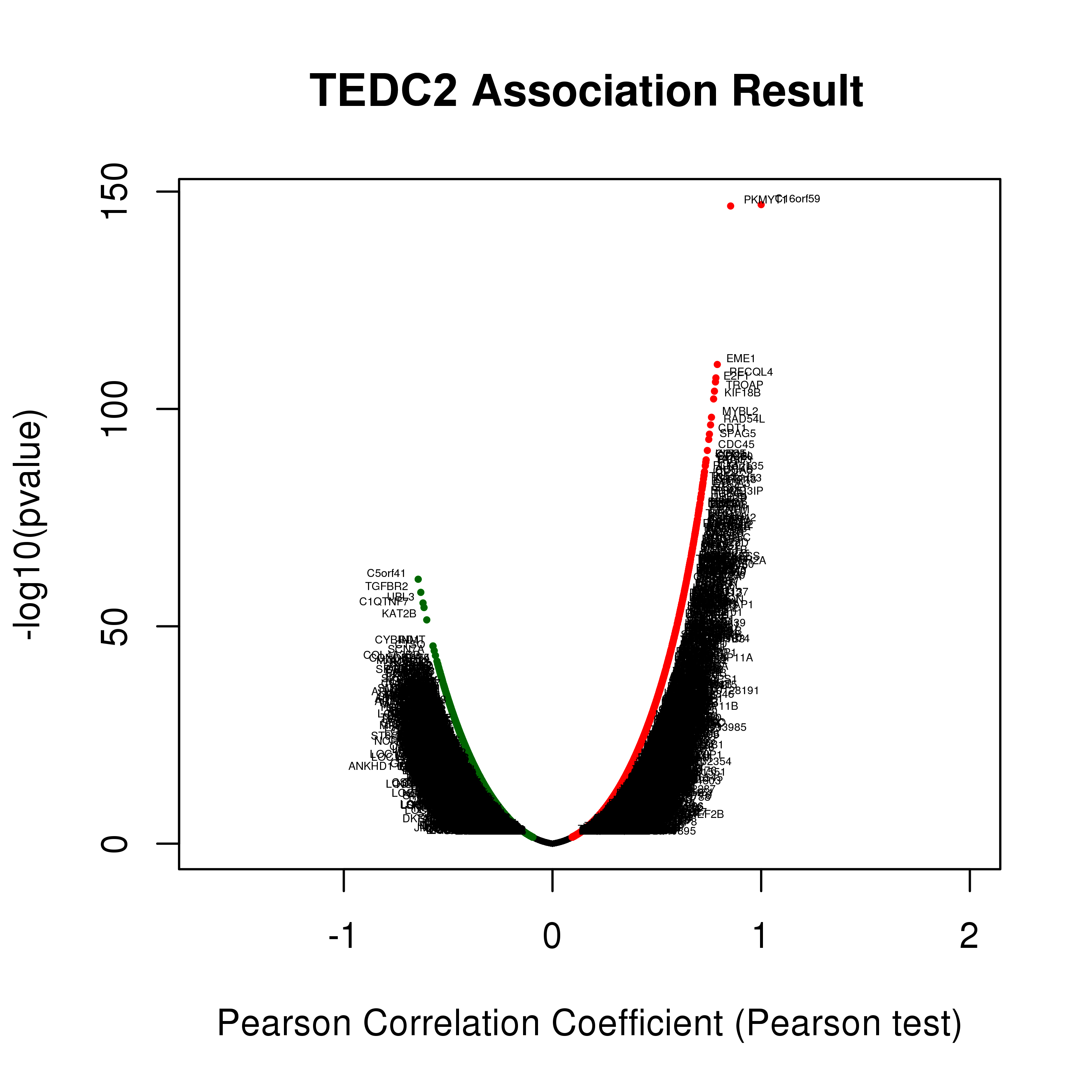

Supplement: Supplementary file 1 — Supplementary Figure S1. [file 41598_2023_32238_MOESM1_ESM.jpg]
