# Supplementary material for: TEDC2 correlated with prognosis and immune microenvironment in lung adenocarcinoma
Source: Sci Rep. 2023 Mar 27;13:5006. doi: 10.1038/s41598-023-32238-8 (PMC10042817; doi:10.1038/s41598-023-32238-8)
Supplement: Supplementary file 2 — Supplementary Tables. [file 41598_2023_32238_MOESM2_ESM.docx]

Supplementary table 1. Top 50 genes positively associated with TEDC2

| Genes | Pearson Correlation | P-value | FDR (BH) |
| --- | --- | --- | --- |
| PKMYT1 | 0.853614208 | 2.05E-147 | 2.05E-143 |
| EME1 | 0.789758554 | 5.86E-111 | 3.90E-107 |
| RECQL4 | 0.783047024 | 7.13E-108 | 3.57E-104 |
| E2F1 | 0.780957419 | 6.19E-107 | 2.47E-103 |
| TROAP | 0.776142713 | 8.22E-105 | 2.74E-101 |
| KIF18B | 0.772009178 | 4.96E-103 | 1.42E-99 |
| MYBL2 | 0.761863632 | 8.16E-99 | 2.04E-95 |
| RAD54L | 0.757505511 | 4.56E-97 | 1.01E-93 |
| CDT1 | 0.75205171 | 6.21E-95 | 1.24E-91 |
| SPAG5 | 0.748884075 | 1.02E-93 | 1.85E-90 |
| CDC45 | 0.742099667 | 3.53E-91 | 5.89E-88 |
| KIFC1 | 0.73617335 | 5.04E-89 | 7.76E-86 |
| CCNF | 0.736080019 | 5.45E-89 | 7.78E-86 |
| ORC6L | 0.734901355 | 1.44E-88 | 1.92E-85 |
| CDC20 | 0.734412664 | 2.15E-88 | 2.68E-85 |
| TACC3 | 0.732408462 | 1.10E-87 | 1.29E-84 |
| TRAIP | 0.732263589 | 1.24E-87 | 1.37E-84 |
| PLK1 | 0.728448989 | 2.66E-86 | 2.80E-83 |
| C1orf135 | 0.72816814 | 3.33E-86 | 3.33E-83 |
| AURKB | 0.726211435 | 1.57E-85 | 1.50E-82 |
| CDCA5 | 0.7254459 | 2.87E-85 | 2.61E-82 |
| KIF2C | 0.723515695 | 1.30E-84 | 1.13E-81 |
| TK1 | 0.72100631 | 9.14E-84 | 7.62E-81 |
| C17orf53 | 0.720206567 | 1.69E-83 | 1.35E-80 |
| CHTF18 | 0.719090428 | 3.99E-83 | 3.05E-80 |
| ESPL1 | 0.719046677 | 4.12E-83 | 3.05E-80 |
| EXO1 | 0.71783012 | 1.04E-82 | 7.45E-80 |
| CDCA3 | 0.716338763 | 3.23E-82 | 2.23E-79 |
| GTSE1 | 0.713343414 | 3.07E-81 | 2.04E-78 |
| BIRC5 | 0.711119218 | 1.60E-80 | 1.03E-77 |
| PSMC3IP | 0.710897503 | 1.89E-80 | 1.18E-77 |
| TUBG1 | 0.70946452 | 5.42E-80 | 3.28E-77 |
| UBE2S | 0.706426756 | 4.97E-79 | 2.92E-76 |
| HJURP | 0.705078814 | 1.32E-78 | 7.52E-76 |
| FEN1 | 0.70302854 | 5.74E-78 | 3.18E-75 |
| UBE2T | 0.702653766 | 7.50E-78 | 4.05E-75 |
| NUF2 | 0.701895069 | 1.29E-77 | 6.77E-75 |
| CDC6 | 0.701290036 | 1.98E-77 | 1.01E-74 |
| ZWINT | 0.700869327 | 2.66E-77 | 1.32E-74 |
| CDCA8 | 0.700851318 | 2.70E-77 | 1.32E-74 |
| FOXM1 | 0.699056909 | 9.57E-77 | 4.55E-74 |
| CENPM | 0.697181018 | 3.56E-76 | 1.65E-73 |
| ORC1L | 0.694342525 | 2.55E-75 | 1.16E-72 |
| TPX2 | 0.694178607 | 2.85E-75 | 1.27E-72 |
| ASF1B | 0.692390039 | 9.72E-75 | 4.23E-72 |
| MCM10 | 0.692003878 | 1.27E-74 | 5.38E-72 |
| C15orf42 | 0.691145379 | 2.27E-74 | 9.46E-72 |
| CENPA | 0.689857398 | 5.45E-74 | 2.22E-71 |
| FAM54A | 0.688553418 | 1.31E-73 | 5.25E-71 |

Supplementary table 2. Top 50 genes negatively associated with TEDC2

| Genes | Pearson Correlation | P-value | FDR (BH) |
| --- | --- | --- | --- |
| C5orf41 | -0.643546113 | 1.49E-61 | 2.66E-59 |
| TGFBR2 | -0.630976966 | 1.54E-58 | 2.48E-56 |
| UBL3 | -0.620351829 | 4.25E-56 | 6.12E-54 |
| C1QTNF7 | -0.615626073 | 4.84E-55 | 6.49E-53 |
| KAT2B | -0.602530619 | 3.31E-52 | 3.82E-50 |
| INMT | -0.572676538 | 3.27E-46 | 3.02E-44 |
| CYBRD1 | -0.572508938 | 3.52E-46 | 3.24E-44 |
| CTSO | -0.56682089 | 4.16E-45 | 3.70E-43 |
| SCN7A | -0.560786983 | 5.43E-44 | 4.66E-42 |
| COL4A3BP | -0.553736089 | 1.02E-42 | 8.25E-41 |
| TLR5 | -0.55051622 | 3.82E-42 | 3.01E-40 |
| FAT4 | -0.549864067 | 4.98E-42 | 3.89E-40 |
| CCNDBP1 | -0.549642735 | 5.45E-42 | 4.24E-40 |
| MAMDC2 | -0.546908119 | 1.65E-41 | 1.25E-39 |
| FCHO2 | -0.545173942 | 3.30E-41 | 2.45E-39 |
| ROBO2 | -0.543919029 | 5.44E-41 | 4.03E-39 |
| PJA2 | -0.541274219 | 1.55E-40 | 1.13E-38 |
| RAI2 | -0.540366973 | 2.22E-40 | 1.61E-38 |
| CTDSPL | -0.539379733 | 3.27E-40 | 2.34E-38 |
| RBL2 | -0.53869013 | 4.28E-40 | 3.04E-38 |
| BTD | -0.538680028 | 4.30E-40 | 3.04E-38 |
| ADH1B | -0.536100798 | 1.17E-39 | 8.12E-38 |
| ADH1A | -0.536005688 | 1.22E-39 | 8.40E-38 |
| DPYSL2 | -0.535108836 | 1.72E-39 | 1.18E-37 |
| SPARCL1 | -0.534634248 | 2.07E-39 | 1.41E-37 |
| CREBL2 | -0.53363082 | 3.05E-39 | 2.06E-37 |
| MGP | -0.532193478 | 5.29E-39 | 3.51E-37 |
| IL33 | -0.532064653 | 5.56E-39 | 3.67E-37 |
| ABCA8 | -0.530750448 | 9.18E-39 | 5.94E-37 |
| PIK3R1 | -0.529992431 | 1.22E-38 | 7.90E-37 |
| CX3CR1 | -0.529219387 | 1.64E-38 | 1.05E-36 |
| CD59 | -0.529080457 | 1.73E-38 | 1.10E-36 |
| FCER1A | -0.528858962 | 1.88E-38 | 1.19E-36 |
| FBXL3 | -0.527208045 | 3.51E-38 | 2.19E-36 |
| SNX30 | -0.524485661 | 9.74E-38 | 5.97E-36 |
| GRIA1 | -0.521830991 | 2.61E-37 | 1.58E-35 |
| SPATA18 | -0.521808876 | 2.63E-37 | 1.59E-35 |
| SCN4B | -0.520603596 | 4.10E-37 | 2.43E-35 |
| FRY | -0.519571872 | 6.00E-37 | 3.52E-35 |
| MTMR10 | -0.519498822 | 6.16E-37 | 3.60E-35 |
| NFIX | -0.518791607 | 7.98E-37 | 4.64E-35 |
| CRY2 | -0.518619778 | 8.50E-37 | 4.93E-35 |
| CHD9 | -0.517867405 | 1.12E-36 | 6.45E-35 |
| CD302 | -0.517197156 | 1.43E-36 | 8.14E-35 |
| HNMT | -0.516621485 | 1.76E-36 | 9.97E-35 |
| C7 | -0.5152422 | 2.90E-36 | 1.63E-34 |
| ITM2B | -0.515123967 | 3.03E-36 | 1.69E-34 |
| CD300LG | -0.514566863 | 3.71E-36 | 2.04E-34 |
| CAT | -0.514227285 | 4.19E-36 | 2.29E-34 |
| SETDB2 | -0.514170907 | 4.28E-36 | 2.34E-34 |
